# Insights into the structure of the CCR4-NOT complex by electron microscopy

**DOI:** 10.1016/j.febslet.2011.05.071

**Published:** 2011-07-21

**Authors:** Fariborz Nasertorabi, Claire Batisse, Meikel Diepholz, Dietrich Suck, Bettina Böttcher

**Affiliations:** a European Molecular Biology Laboratory, (EMBL), Meyerhofstrasse 1, D-69117 Heidelberg, Germany; b University of Edinburgh, School of Biological Sciences, King’s Buildings, Mayfield Road, Edinburgh EH9 3JR, UK

**Keywords:** CCR4-NOT complex, Electron microscopy, Image processing, Complex purification, Deadenylation

## Abstract

The CCR4-NOT complex is a deadenylation complex, which plays a major role for mRNA stability. The complex is conserved from yeast to human and consists of nine proteins NOT1–NOT5, CCR4, CAF1, CAF40 and CAF130. We have successfully isolated the complex using a Protein A tag on NOT1, followed by cross-linking on a glycerol gradient. All components of the complex were identified by mass spectrometry. Electron microscopy of negatively stained particles followed by image reconstruction revealed an L-shaped complex with two arms of similar length. The arms form an accessible cavity, which we think could provide an extensive interface for RNA-deadenylation.

**Structured summary of protein interactions:**

**CAF1** physically interacts with **CCR4** and **NOT1** by tandem affinity purification (View interaction)

## Highlights

► The CCR4-NOT complex was purified from yeast. ► The complex was stabilized by cross-linking. ► Electron microscopic studies revealed that the complex is L-shaped. ► The arms of the complex form an accessible cavity.

## 1 Introduction

Gene expression in eukaryotic cells progresses through different steps, which are tightly regulated to ensure the integrity of the transcriptome. After transcription the 3′-end of the mRNA is adenylated with a poly-A-tail, which is important for transport to the cytoplasm and essential for offsetting mRNA-degradation. Adenylation is counterbalanced by deadenylation, which removes the poly-A-tail and thus enables the degradation of the mRNA in the cytoplasm. Poly (A) deadenylation is catalyzed by the CCR4-NOT complex, which is conserved from yeast to human [1–3]. In yeast the complex occurs in two different sizes with approximate masses of 1.0 MDa and 1.9 MDa [4–6]. The complex consists of at least nine core subunits, namely CCR4, CAF1, CAF40, CAF130 and NOT1–5 [2,4,5,7,8]. Of these proteins only NOT1 is essential for viability [9]. Except for CAF40 and CAF130 all other proteins were initially identified by genetic selection for transcriptionally regulating elements [10], which suggests that the complex plays a global regulatory role in gene expression.

The CCR4-NOT-complex contains CCR4 and CAF1, which are the major mRNA deadenylases in yeast [11,12]. Both proteins exert a 3′–5′ exonulclease activity for mRNA, with a preference for poly A [11,13]. Despite structures of both proteins from different species are known [14–17] it remains unclear why two different deadenylases are incorporated in the same complex. Furthermore, structural insights into the organization of the whole complex are still missing. Here we show an effective method for purifying a homogeneous complex, which we have studied by electron microscopy and image reconstruction. These investigations give a first glimpse of the three-dimensional structure of the CCR4-NOT-complex from yeast.

## 2 Materials and methods

Yeast strain MATa *ade*2 *arg*4 *leu*2-3, 112 *trp1*-289 *ura*3-5 (A kind gift from Anne-Claude Gavin, EMBL-Heidelberg) with a TAP-tag [18] fused either to the N-terminus of CAF1 or NOT1 was used.

### 2.1 Cell growth

A single colony of a freshly stroke plate was used to inoculate 20 ml YPD media (2% yeast extract, 1% peptone, 2% glucose) with additional 3% glucose for overnight growth at 30 °C. 750 ml of YPD media were inoculated with the overnight cell culture and let it grow overnight until OD = 7–9. Cells were harvested and centrifuged for 15 min at 5000×*g*. Typically, 12 g cell pellet/L of cell culture was obtained. The pellet was washed with distilled water, flash frozen in liquid nitrogen and stored at −80 °C.

### 2.2 Purification and preparation for electron microscopy analysis

All the steps have been carried out in 4 °C unless indicated: 150 g of cells were thawed in 240 ml of lysis buffer (200 mM Tris HCl pH 7.7, 600 mM Potassium acetate, 10% glycerol, 2 mM Benzamidine, 2 μM Leupeptin, 2 μM Pepstatin A, 4 μM Chymostatin, 3 μM Aprotinin, 1.5 μM Antipain, 1 mM Pefablock SC, and two tablets of complete protease inhibitor cocktail EDTA free (Roche)). Cells were disrupted with glass beads in lysis buffer using a Pulverisette (Fritsch, 3 cycles milling with power set to 350 for 4 min, 1 min break between cycles) and the lysate was diluted with lysis buffer containing 40% glycerol to have a final concentration of 20% glycerol. The cell lysate was centrifuged at 35 000×*g* for 45 min. The supernatant was centrifuged at 170 000×*g* for 1.5 h prior to incubation with 100 μl of IgG sepharose 6 fast flow (GE Healthcare, USA), which had been pre-equilibrated with the lysis buffer without protease inhibitors, for 2 h on a turning wheel. Afterwards, the beads were transferred into a MoBiTech column (Mobicol, 35 μm filter) and washed with 25 ml of lysis buffer, 25 ml of wash buffer 1 (200 mM Tris HCl pH 7.7, 600 mM Potassium acetate, 0.5 mM DTT,10% glycerol), 25 ml of wash buffer 2 (200 mM Tris HCl pH 7.7, 600 mM Potassium acetate, 0.5 mM DTT, 10% glycerol, 0.01 % NP-40), 25 ml of wash buffer 3 (200 mM Tris HCl pH 7.7, 600 mM Potassium acetate, 0.5 mM DTT) and 10 ml of TEV cleaving buffer (50 mM HEPES pH 7.7, 150 mM NaCl, 0.5 mM DTT). Then the sample was incubated with 35 μg of TEV protease for 2 h at 16 °C on a turning wheel. The cleaved sample was eluted by adding 500 μl of TEV cleaving buffer and was followed by a calmodulin binding step [18]. Alternatively, the TEV-cleaved, eluted sample was cross-linked by glutaraldehyde (Electron Microscopy Sciences) on a glycerol gradient (Grafix protocol; [19]). The gradient fractions were tested by dot blot using an anti TAP antibody (CAB 1001, Open biosystem, USA), to identify the tagged protein. The fraction showing the strongest signal in dot-blot was prepared by sandwich negative staining using 2% uranyl acetate [20]. For evaluating the effect of cross-linking, the TEV-eluted sample was also purified on a glycerol gradient without added glutaraldehyde; followed by dot-blot and electron microscopy as described above.

### 2.3 Blue native gel electrophoresis

The most concentrated fraction of the glycerol gradient without added glutaraldehyde (non-cross-linked sample) was loaded onto a Native PAGE Novex 3–12% Bis–tris gel (1.0 mm, Invitrogen) followed by electrophoresis according to manufacturers’ instructions.

### 2.4 Electron microscopy

Micrographs were recorded under low dose conditions at a nominal magnification of 27 500 with an accelerating voltage of 200 kV using a Philips CM200 FEG equipped with a 2 k × 2k CCD-camera (TVIPS GmbH). The pixel size was 5.16 Å. For random conical tilt reconstruction (RCT, [21]) pairs of micrographs were recorded, with the sample tilted to –55° (first micrograph) and 0° (second micrograph). 156 pairs of micrographs were used for further analysis.

### 2.5 Image processing

7030 pairs of particles from tilted and untilted samples were selected using Spider WEB [22]. Tilted particles were centered by translational alignment to mass centered class averages using IMAGIC [23]. Untilted particles were rotationally and translationally aligned and classified. Alignment and classification were repeated several times, using the current best class averages as references.

We calculated a RCT for each class using Spider [22]. Several of these RCTs were used as reference to start maximum likelihood 3D-sorting [24] of 1000 class averages of untilted particle images into five volumes. The process converged to similar volumes irrespective of the selected starting volume. Finally, one of the volumes, which represented most of the class averages, was used for iterative refinement of the spatial orientations of the tilted and untilted particles by projection matching against projections of the current best map using Spider. Three-dimensional maps were calculated using back-projection interpolated in Fourier space.

A Fourier–Shell correlation (FSC) between two maps that were calculated from different subsets of particle images, was determined. The resolution was estimated using the spatial frequency were the FSC dropped to 0.5 or 0.14, respectively.

Maps were displayed with Chimera [25] and segmented with the Segger-option for estimating volumes of the sub-domains.

## 3 Results and discussion

**Fig. 1:**
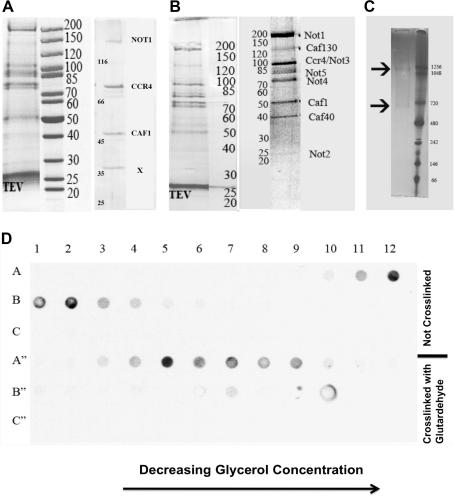
Biochemical characterization of the purification of the CCR4-NOT complex. (A) Coomassie stained SDS-gels of tandem affinity purification with CAF1 as bait after TEV cleavage (left) and after calmodulin purification (right). (B) Coomassie stained SDS-gels of tandem affinity purification with NOT1 as bait after TEV cleavage (left) and after TEV-cleavage followed by a glycerol gradient (right). (C) Blue Native gel (3–12%) of the non-cross-linked CCR4-NOT complex recovered from the glycerol gradient (left) and molecular weight markers (right). The complex forms two species (arrows). (D) Dot blot assay of fractions of the glycerol gradient using an anti-TAP antibody to identify NOT1. Fractions in A–C refer to the non-cross-linked complex and in A′–C′ to the cross-linked complex. Fraction 1 is at the bottom of the gradient and has the highest glycerol and glutaraldehyde (cross-linked complex only) concentration.

The CCR4-NOT complex was purified according to a modified TAP-protocol using both affinity tags of the TAP-tag on the N-terminus of CAF1. This yielded a complex that consisted of three components, which we identified by mass spectrometry as NOT1, CAF1 and CCR4 (Fig. 1A, right panel). However, several other subunits, which were previously identified as being part of the CCR4-NOT-complex (NOT2–5, CAF130, CAF40) were only present after the TEV cleavage (Fig. 1A, left panel), but were absent after the calmoduline purification (Fig. 1A, right panel). Especially, most of the subunits of the NOT-subcomplex were lost and the band of the NOT1 protein was relatively weak, indicating a recovery of NOT1 in sub-stoichiometric amounts. Therefore, we changed the bait to the NOT1-protein, which is the scaffolding protein for the NOT-subcomplex. Similar to the tag on CAF1, the purification yielded a complex where most of the proteins were present after the TEV cleavage (Fig.1B, left panel) but with an increased amount of recovered complex (not shown). Next, we considered that binding of this complex to the Calmodulin beads followed by chelating of Calcium ions with EGTA might interfere with the complex integrity. Therefore, we replaced the calmodulin purification with a glycerol gradient that is much milder. This yielded a larger complex, which contained all the expected nine components of the CCR4-NOT-complex, namely NOT1–5, CCR4, CAF1, CAF40 and CAF130 (Fig. 1B, right panel).

**Fig. 2:**
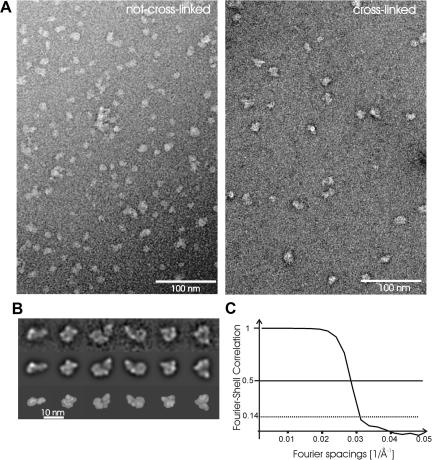
Electron microscopic analysis of the CCR4-NOT complex. (A) Micrographs of negatively stained CCR4-NOT-complex without cross-linking (left) and with cross-linking (right). (B) Two-dimensional averages of the CCR4-NOT complex (top row) with matching projections of the three-dimensional map (central row) and surface presentations of the map in the same orientation as the projections (bottom row). (C) Fourier–Shell Correlation between two maps calculated from different subsets of particle images.

Electron microscopy of negatively stained samples showed a large complex with some heterogeneity in size (Fig. 2A, left panel). This heterogeneity was confirmed by native gels, which showed two major bands of 1.0 MDa and 0.7 MDa (Fig. 1C, arrows). The fact that the two species detected in the native gel, originated from the same fraction of the glycerol gradient, suggested an inherent instability of the complex. In order to stabilize the complex for further structural studies, the sample was cross-linked with glutaraldehyde (GraFix protocol; [19]). The fractions containing NOT1 were identified with a dot-blot-assay against the TAP-tag at NOT1 (Fig. 1D). The blot showed that the NOT1-containing cross-linked complex had shifted towards higher glycerol concentrations, indicating the recovery of a somewhat larger complex. Electron microscopy of negatively stained samples confirmed that the cross-linked CCR4-NOT-complex was more homogeneous than the non-cross-linked complex. Furthermore, it was virtually free of random aggregates implying that the shift was not due to random-aggregation.

**Fig. 3:**
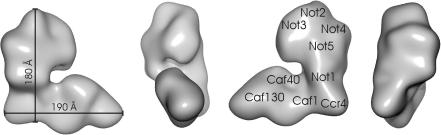
Surface representation of the three-dimensional map. The tentative positions of the subunits are indicated. The positions are mainly based on size considerations and require further experimental validation.

After cross-linking, the homogeneity was sufficient for further structural analysis by electron microscopy and image processing. By using RCT [21,22] maximum likelihood sorting [24] and projection matching [22], we could determine a three-dimensional map of a typical conformation of the 9-subunit complex. Projections of this map matched with 2D-averages calculated from particles that were assigned with the same Euler angles (Fig. 2B) highlighting the validity of the map. The resolution was 33 Å at FSC = 0.5 or 31 Å at FSC = 0.14 (Fig. 2C). The map revealed a flat, L-shaped particle (Fig. 3) with two arms of similar length (180 Å and 190 Å). The shorter arm accounted for ca. 600 kDa, whereas the longer, thinner arm accounted for ca. 300 kDa and the hinge domain for approximately 100 KDa. Consequently, the shorter arm together with the hinge would be large enough to account for the 700 kDa complex that we observed in native gels. Considering that NOT1 was used as bait, it is likely, that the 700 kDa sub-complex contained the NOT1-protein.

The relative arrangement of both arms varied between different reconstructions indicating flexibility of the whole assembly and explaining the limited resolution of the map. Both arms form an accessible cavity in their centre, which could provide an extensive platform for controlled interaction with RNA and accessory regulating factors.

The current knowledge on binary protein-protein interactions and the lack of high-resolution structures of individual subunits and sub-complexes leave ambiguities in the interpretation of the map of the CCR4-NOT-complex. Considering that NOT1 is the major scaffolding protein to which the other NOT proteins bind [4] suggests that the shorter arm is mainly formed by the NOT-proteins, which account for ca. 500 kDa. Furthermore, NOT1 is in contact with CAF1, which binds to CCR4 [4]. This could place CCR4 at the strategic position in the hinge-domain, which has a similar shape and dimension as the catalytic domain of the human homologue of CCR4 [17]. It is likely that the remaining CAF40 and CAF130 account for most of the longer arm, which agrees with the lack of direct interactions between CAF40 and CAF130 with the NOT2–5 proteins.

## Accession numbers

The map was deposited in the EMDB (ID 1901).

## References

[1] Albert T.K., Lemaire M., van Berkum N.L., Gentz R., Collart M.A., Timmers H.T. (2000). Isolation and characterization of human orthologs of yeast CCR4-NOT complex subunits. Nucleic Acids Res..

[2] Draper M.P., Salvadore C., Denis C.L. (1995). Identification of a mouse protein whose homolog in Saccharomyces cerevisiae is a component of the CCR4 transcriptional regulatory complex. Mol. Cell Biol..

[3] Gavin A.C. (2002). Functional organization of the yeast proteome by systematic analysis of protein complexes. Nature.

[4] Bai Y., Salvadore C., Chiang Y.C., Collart M.A., Liu H.Y., Denis C.L. (1999). The CCR4 and CAF1 proteins of the CCR4-NOT complex are physically and functionally separated from NOT2, NOT4, and NOT5. Mol. Cell Biol..

[5] Liu H.Y., Badarinarayana V., Audino D.C., Rappsilber J., Mann M., Denis C.L. (1998). The NOT proteins are part of the CCR4 transcriptional complex and affect gene expression both positively and negatively. Embo J..

[6] Liu H.Y. (2001). Characterization of CAF4 and CAF16 reveals a functional connection between the CCR4-NOT complex and a subset of SRB proteins of the RNA polymerase II holoenzyme. J. Biol. Chem..

[7] Chen J., Rappsilber J., Chiang Y.C., Russell P., Mann M., Denis C.L. (2001). Purification and characterization of the 1.0 MDa CCR4-NOT complex identifies two novel components of the complex. J. Mol. Biol..

[8] Draper M.P., Liu H.Y., Nelsbach A.H., Mosley S.P., Denis C.L. (1994). CCR4 is a glucose-regulated transcription factor whose leucine-rich repeat binds several proteins important for placing CCR4 in its proper promoter context. Mol. Cell Biol..

[9] Maillet L., Tu C., Hong Y.K., Shuster E.O., Collart M.A. (2000). The essential function of Not1 lies within the Ccr4-Not complex. J. Mol. Biol..

[10] Collart M.A. (2003). Global control of gene expression in yeast by the Ccr4-Not complex. Gene.

[11] Daugeron M.C., Mauxion F., Seraphin B. (2001). The yeast POP2 gene encodes a nuclease involved in mRNA deadenylation. Nucleic Acids Res..

[12] Tucker M., Valencia-Sanchez M.A., Staples R.R., Chen J., Denis C.L., Parker R. (2001). The transcription factor associated Ccr4 and Caf1 proteins are components of the major cytoplasmic mRNA deadenylase in Saccharomyces cerevisiae. Cell.

[13] Chen J., Chiang Y.C., Denis C.L. (2002). CCR4, a 3′–5′ poly(A) RNA and ssDNA exonuclease, is the catalytic component of the cytoplasmic deadenylase. Embo J..

[14] Horiuchi M. (2009). Structural basis for the antiproliferative activity of the Tob-hCaf1 complex. J. Biol. Chem..

[15] Jonstrup A.T., Andersen K.R., Van L.B., Brodersen D.E. (2007). The 1.4-A crystal structure of the S. pombe Pop2p deadenylase subunit unveils the configuration of an active enzyme. Nucleic Acids Res..

[16] Thore S., Mauxion F., Seraphin B., Suck D. (2003). X-ray structure and activity of the yeast Pop2 protein: a nuclease subunit of the mRNA deadenylase complex. EMBO Rep..

[17] Wang H. (2010). Crystal structure of the human CNOT6L nuclease domain reveals strict poly(A) substrate specificity. Embo J..

[18] Puig O., Caspary F., Rigaut G., Rutz B., Bouveret E., Bragado-Nilsson E., Wilm M., Seraphin B. (2001). The tandem affinity purification (TAP) method: a general procedure of protein complex purification. Methods.

[19] Kastner B. (2008). GraFix: sample preparation for single-particle electron cryomicroscopy. Nat. Methods.

[20] Diepholz M. (2008). A different conformation for EGC stator subcomplex in solution and in the assembled yeast V-ATPase: possible implications for regulatory disassembly. Structure.

[21] Radermacher M. (1988). Three-dimensional reconstruction of single particles from random and nonrandom tilt series. J. Electron Microsci. Tech..

[22] Frank J., Radermacher M., Penczek P., Zhu J., Li Y., Ladjadj M., Leith A. (1996). SPIDER and WEB: processing and visualization of images in 3D electron microscopy and related fields. J. Struct. Biol..

[23] van Heel M., Harauz G., Orlova E.V., Schmidt R., Schatz M. (1996). A new generation of the IMAGIC image processing system. J. Struct. Biol..

[24] Scheres S.H., Gao H., Valle M., Herman G.T., Eggermont P.P., Frank J., Carazo J.M. (2007). Disentangling conformational states of macromolecules in 3D-EM through likelihood optimization. Nat. Methods.

[25] Pettersen E.F., Goddard T.D., Huang C.C., Couch G.S., Greenblatt D.M., Meng E.C., Ferrin T.E. (2004). UCSF Chimera–a visualization system for exploratory research and analysis. J. Comput. Chem..

